# Transitioning a digital health innovation from research to routine practice: Two-way texting for male circumcision follow-up in Zimbabwe

**DOI:** 10.1371/journal.pdig.0000066

**Published:** 2022-06-15

**Authors:** Phiona Marongwe, Beatrice Wasunna, Jacqueline Gavera, Vernon Murenje, Farai Gwenzi, Joseph Hove, Christine Mauhy, Sinokuthemba Xaba, Raymond Mugwanya, Batsirai Makunike-Chikwinya, Tinashe Munyaradzi, Michael Korir, Femi Oni, Antony Khaemba, Mourice Barasa, Marrianne Holec, Vuyelwa Sidile-Chitimbire, Mufuta Tshimanga, Isaac Holeman, Scott Barnhart, Caryl Feldacker

**Affiliations:** 1 Zimbabwe Technical Training and Education Centre for Health (Zim-TTECH), Harare Zimbabwe; 2 Department of Global Health, University of Washington, Seattle, Washington, United States of America; 3 Medic, Nairobi, Kenya; 4 Zimbabwe Association of Church related Hospitals (ZACH), Harare Zimbabwe; 5 Zimbabwe Community Health Intervention Research (ZiCHIRe), Harare Zimbabwe; 6 Ministry of Health and Child Care (MoHCC), Harare Zimbabwe; 7 International Training and Education Center for Health, Department of Global Health, University of Washington, Seattle, Washington, United States of America; 8 Medic, San Francisco, California, United States of America; 9 School of Medicine, University of Washington, Seattle, Washington, United States of America; Vanderbilt University, UNITED STATES

## Abstract

Adult medical male circumcision (MC) is safe: global notifiable adverse event (AE) rates average below 2.0%. With Zimbabwe’s shortage of health care workers (HCWs) compounded by COVID-19 constraints, two-way text-based (2wT) MC follow-up may be advantageous over routinely scheduled in-person reviews. A 2019 randomized control trial (RCT) found 2wT to be safe and efficient for MC follow-up. As few digital health interventions successfully transition from RCT to scale, we detail the 2wT scale-up approach from RCT to routine MC practice comparing MC safety and efficiency outcomes. After the RCT, 2wT transitioned from a site-based (centralized) system to hub-and-spoke model for scale-up where one nurse triaged all 2wT patients, referring patients in need to their local clinic. No post-operative visits were required with 2wT. Routine patients were expected to attend at least one post-operative review. We compare 1) AEs and in-person visits between 2wT men from RCT and routine MC service delivery; and 2) 2wT-based and routine follow-up among adults during the 2wT scale-up period, January to October 2021. During scale-up period, 5084 of 17417 adult MC patients (29%) opted into 2wT. Of the 5084, 0.08% (95% CI: 0.03, 2.0) had an AE and 71.0% (95% CI: 69.7, 72.2) responded to ≥1 daily SMS, a significant decrease from the 1.9% AE rate (95% CI: 0.7, 3.6; p<0.001) and 92.5% response rate (95% CI: 89.0, 94.6; p<0.001) from 2wT RCT men. During scale-up, AE rates did not differ between routine (0.03%; 95% CI: 0.02, 0.08) and 2wT (p = 0.248) groups. Of 5084 2wT men, 630 (12.4%) received telehealth reassurance, wound care reminders, and hygiene advice via 2wT; 64 (19.7%) were referred for care of which 50% had visits. Similar to RCT outcomes, routine 2wT was safe and provided clear efficiency advantages over in-person follow-up. 2wT reduced unnecessary patient-provider contact for COVID-19 infection prevention. Rural network coverage, provider hesitancy, and the slow pace of MC guideline changes slowed 2wT expansion. However, immediate 2wT benefits for MC programs and potential benefits of 2wT-based telehealth for other health contexts outweigh limitations.

## Author summary

Although digital health innovations hold promise to improve patient outcomes and reduce workload, there is very little evidence of success from routine settings in lower income countries. We successfully scaled a two-way texting (2wT) intervention from research to routine practice, providing men with SMS-based telehealth for male circumcision (MC) follow-up rather than requiring in-person reviews. 2wT operates without consistent connectivity, supports providers with decision-making tools, and requires patients to have only basic phones. Like in the research setting, 2wT reduced healthcare worker workload and maintained patient safety when implemented with routine MC teams. The COVID-19 pandemic led added urgency to 2wT expansion. We describe the successes and challenges of the 2wT scale-up, providing lessons learned that may enable others to adapt and adopt this open-source App in other health contexts that could benefit from a similar, low-cost, short-term, telehealth approach.

## Background

By 2020, voluntary medical male circumcision (MC) reached nearly 27 million men in Southern Africa [1,2] with an average moderate and severe adverse event (AE) rate of 0.8% (range: 0.4–8.0) [3–8]. This corresponds to 99% of men healing well. In routine MC programs, men are recommended to have multiple, in-person reviews by a nurse or doctor, ideally on post-operative days 2, 7 and 42, to assess healing progress [9]. Although in-person follow-up rates may be overreported [8,10], global adherence to these visits is reported as high [11]. Requiring multiple post-operative visits where most men heal without complication is highly inefficient, raises unnecessary COVID-19 exposure risks, and increases healthcare worker (HCW) burden.

A 2019 randomized control trial (RCT) led by the International Training and Education Center for Health (I-TECH) at the University of Washington (UW); technology partner, Medic, Nairobi, Kenya; and the ZAZIC consortium (a name comprising letters from Zimbabwe Community Health intervention Research project (ZICHIRE); Zimbabwe Association of Church-related Hospitals (ZACH); and Zimbabwe Technical Assistance Training and Education Centre for Health (Zim-TTECH)) determined that SMS interaction between patients and MC providers for 13 critical post-operative days reduced follow-up workload by 87%, doubled AE ascertainment, and lowered costs by over $2 per patient [12,13] as compared to routine in-person care. HCWs and patients found the system highly usable, and both felt reassured that men could heal independently or seek care when needed [12].

Like many other health services, COVID-19 infection prevention efforts forced a pause in ZAZIC’s MC service provision in Zimbabwe in March 2020. Although precautions were implemented to reduce COVID-19 infection spread during MC surgical procedures, returning for multiple reviews heightened provider and patient COVID-19 risks. To reduce risk of COVID-19 transmission, Zimbabwe’s Ministry of Health and Child Care (MoHCC) approved wide use of 2wT for MC follow-up in February 2021.

We detail transitioning 2wT-based follow-up from RCT to scale-up within ZAZIC’s routine MC services, comparing in-person visits (workload) and AEs between 1) 2wT men from RCT and routine contexts; and 2) routine 2wT and in-person follow-up from January 2021 to October 2021. We describe scale-up successes and challenges to inform MC policy and to increase the sustainability of future digital health innovations.

## Methods

### 2wT program description

#### 2wT Technology

**Fig 1:**
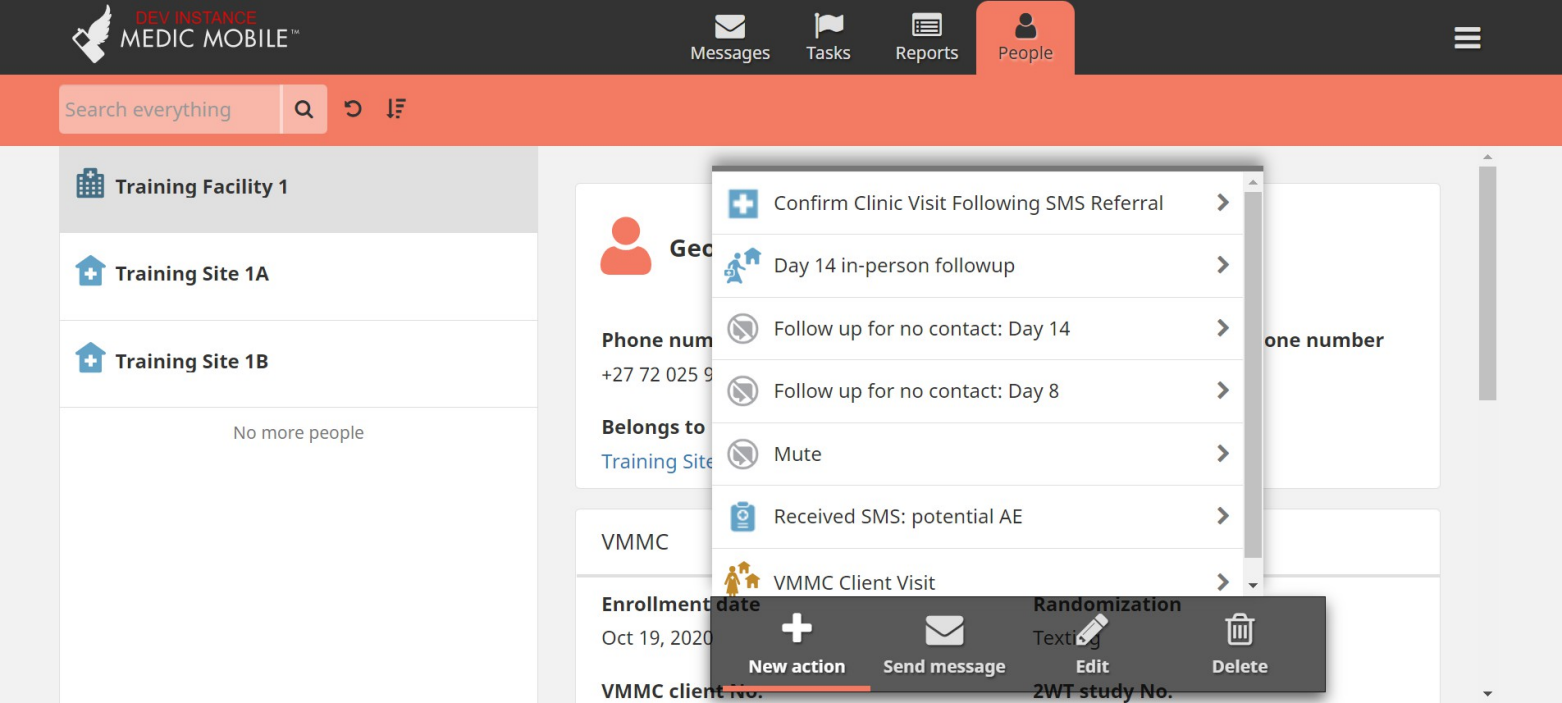
CHT “Person Tab”. This 2wT tab displays options for nurses to complete various 2wT Reports and Tasks for any enrolled patient on the PC or mobile (tablet or phone) app.

**Fig 2:**
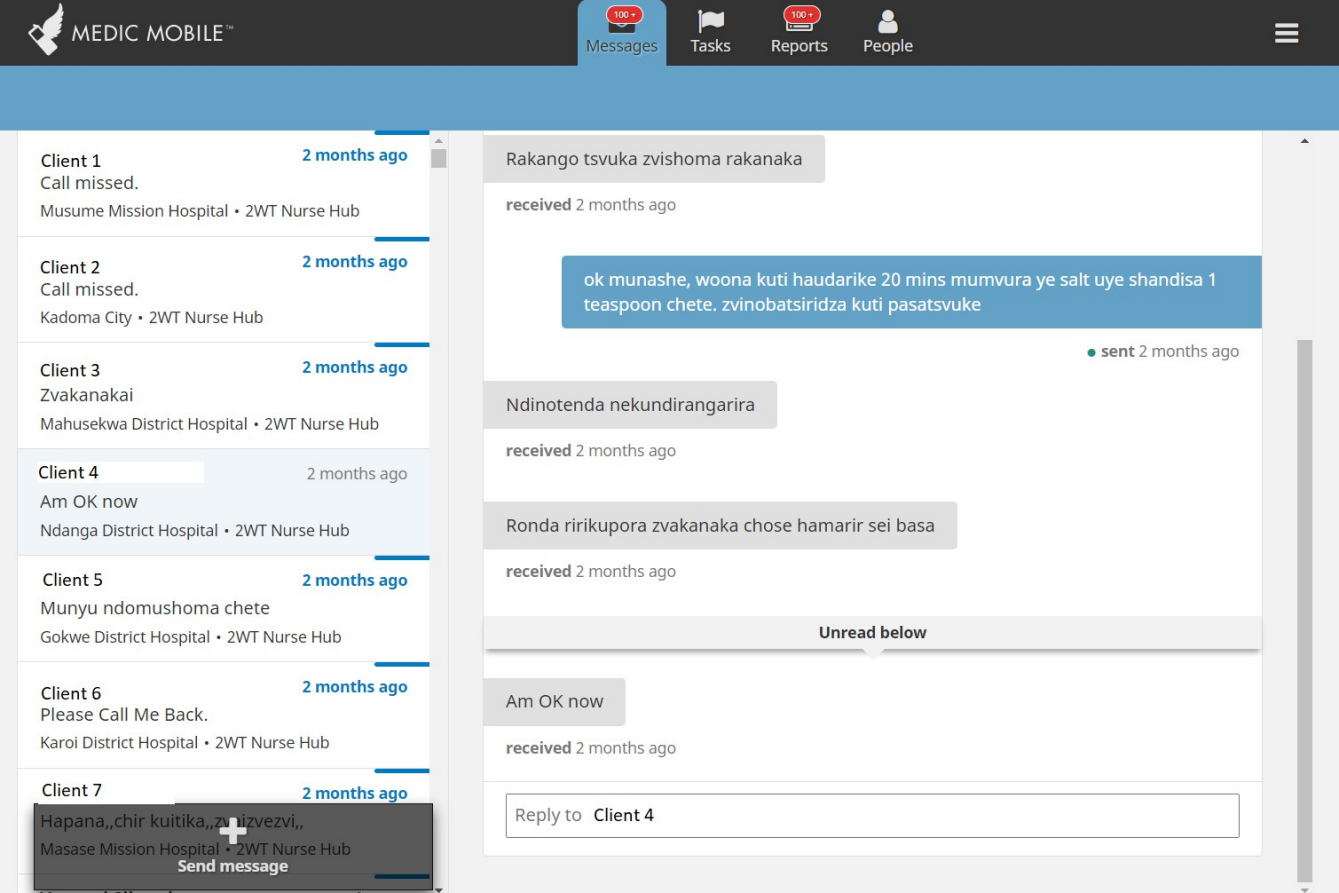
CHT “Messages Tab”. This 2wT tab provides an interface for interactive SMS between 2wT patients and providers on the PC or mobile (tablet or phone) app.

**Fig 3:**
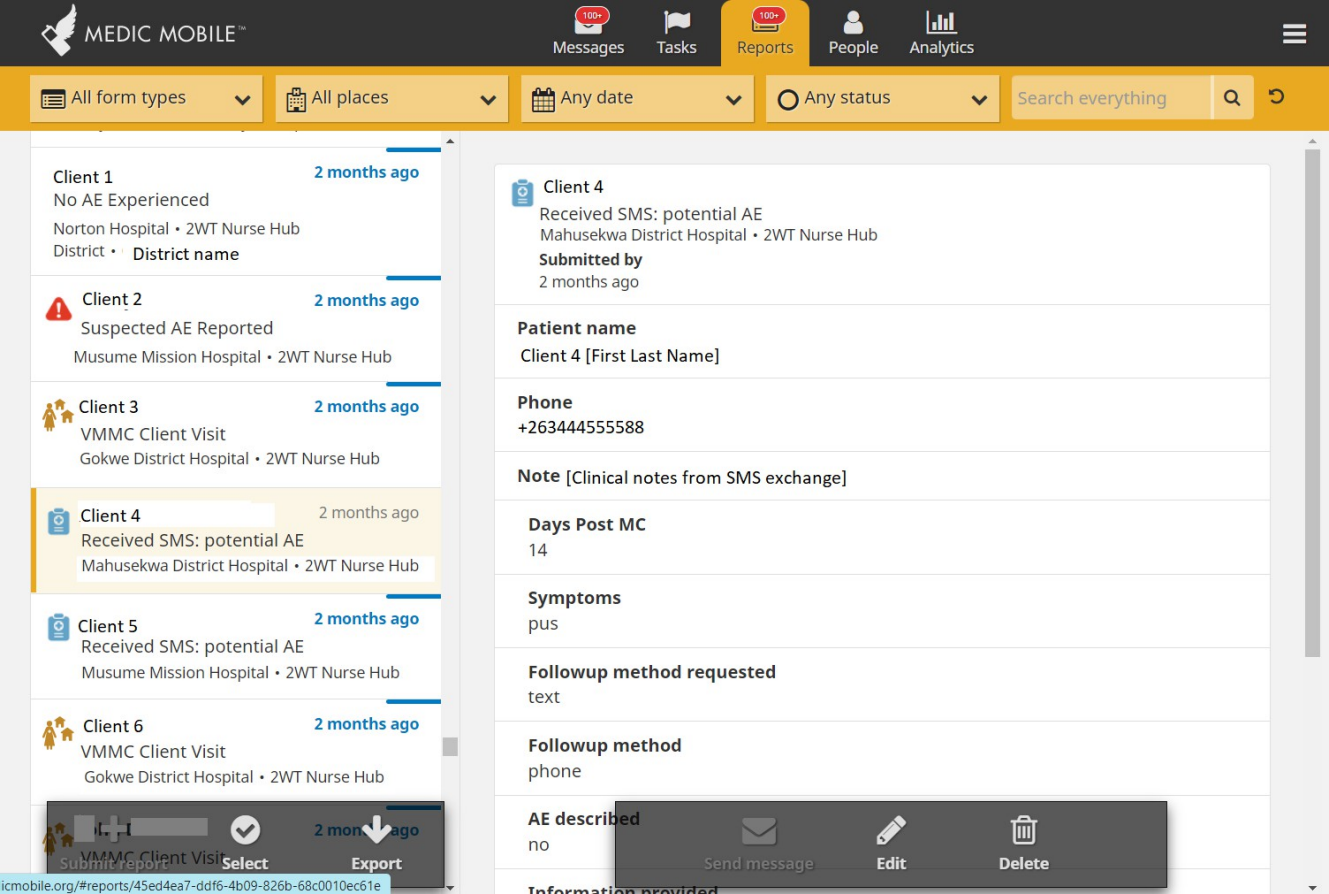
CHT “Reports Tab”. This 2wT tab documents all 2wT interactions, including patient daily responses and nurse follow-up, providing data for routine and 2wT-specific reporting.

Since 2008, Medic (formerly, *Medic Mobile)*, a leader in the global mHealth community, supported almost 40,000 health workers to provide over 60 million caring activities across 25 countries. Medic is the steward of Community Health Toolkit (CHT), an open-source project supporting dozens of mHealth implementations [14–17] and user-centred designed apps, including for 2wT in Zimbabwe [12,18] (Figs 1–3). CHT interventions and apps work with or without internet connectivity, in any language, on basic phones, smartphones, tablets, or computers. CHT apps are tailored to meet 80% of the key digital health characteristics recommended by World Health Organization (WHO) [19] and adhere to Fast Healthcare Interoperability Resources (FHIR) standards. 2wT is based on Medic’s CHT and includes automated and interactive messaging, task management features (incomplete task; message response needed), longitudinal patient records, data collection forms, routine syncing, and dashboards for routine monitoring (e.g., patient response rates, AEs).

#### 2wT transition from RCT to routine care

**Fig 4:**
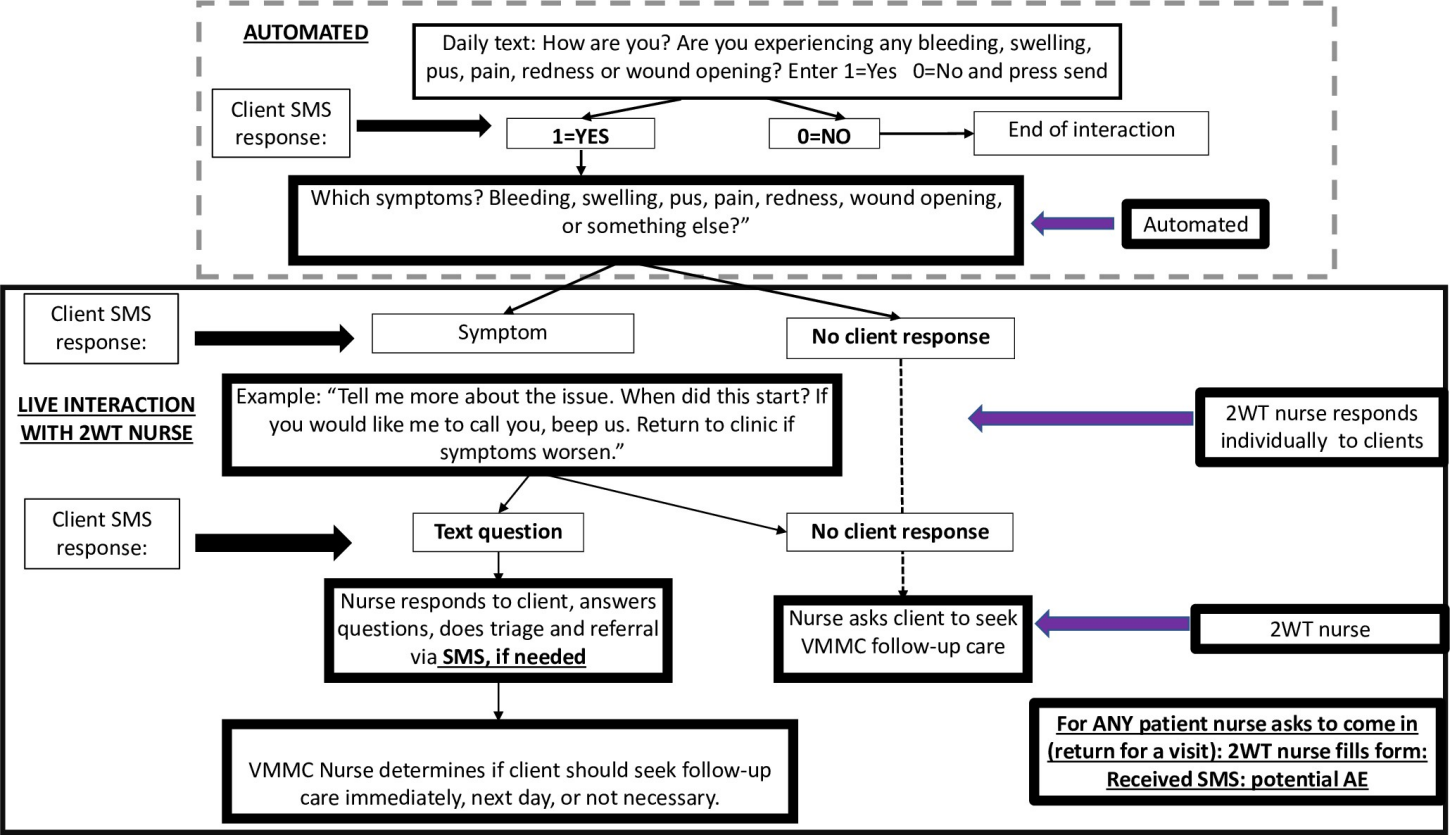
2wT RCT flow chart. Fig 4 flow displays hybrid 2wT, with both automated and interactive (manual) SMS, interaction between patients and providers in a centralized model where a single site’s clinical provider(s) manages 2wT patients enrolled at that specific site.

**Fig 5:**
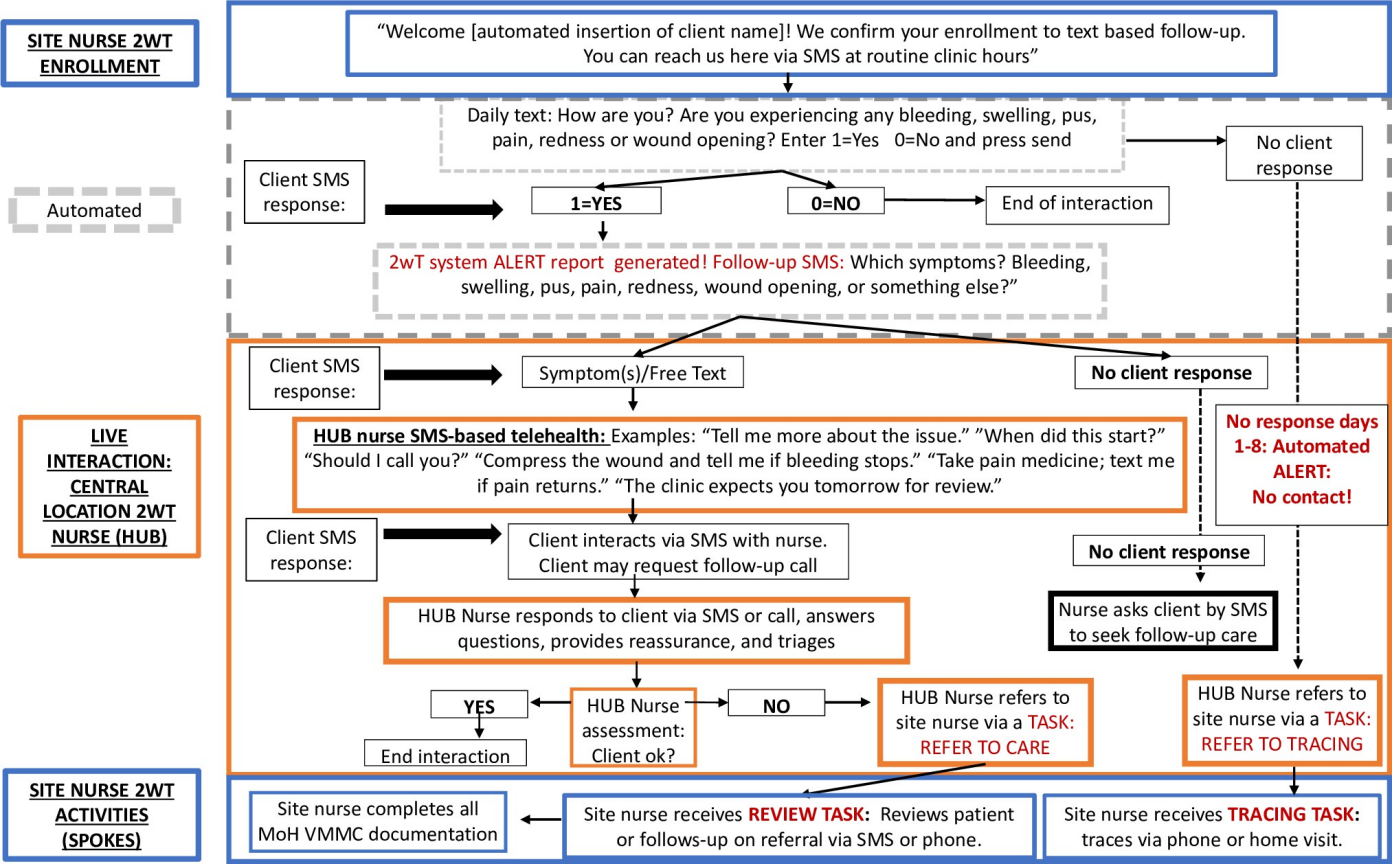
2wT scale-up flow chart. Fig 5 flow displays hybrid 2wT, with both automated and interactive (manual) SMS, interaction between patients and providers in a hub-and-spoke model where a Hub clinical provider(s) manages 2wT patients from multiple 2wT enrolment sites.

ZAZIC conducted a highly participatory design meeting with Medic and user stakeholders (MoHCC, other MC implementing partners) to guide HCW standard operating procedure (SOP) changes from RCT to routine settings (Table 1). SOPs from the meeting reflected human resource training needs and plans for overcoming electricity, cell service and network coverage challenges for scale up. For RCT, patient messaging, tracing, and in-person clinical reviews were completed by the 2wT nurse at the study sites using a centralized model (Fig 4). For scale-up, one 2wT nurse (Hub nurse) was stationed at MoHCC to provide system-wide oversight and quality assurance as part of national MC program while other 2wT-based activities occurred at site (facility) level. Additional RCT implementation details were previously published [12,18]. A 3-tier system was developed to take 2wT from RCT to scale (Fig 5).

**Table 1:** Comparison of MC procedures: routine follow-up; 2wT in RCT; and 2wT in routine settings.

MC service component	Routine follow-up	2wT: RCT	2wT: Routine
**Day 0 activities–Day of MC**			
**RCT consent for 2wT**	X	X	
**Routine MC registration and patient intake forms**	X	X	X
**MC surgery and counselling**	X	X	X
**Counselling on signs of potential AEs**	X	X	X
**Bandage removal instructions**		X	X
**2wT messaging overview**		X	X
**2wT enrolment on Android phones**			X
**Scheduled in-person follow-up**			
**Routine Day-2**	X		
**Routine Day-7**	X		
**RCT-specific Day-14**	X	X	
**Routine Day-42**	X		
**Daily texts on post-operative days 1–13**		X	X
**Routine lost to follow-up tracing by site team**			
**Day 2**	X	X	
**Day 7**	X	X	X
**MoHCC standard AE procedures**			
**In-person, any day, follow-up for suspicion of AE**	X	X	X
**Emergency MC after-hours care**	X	X	X
**AE identification**	X	X	X
**AE severity grading**	X	X	X
**AE management and treatment**	X	X	X
**Site-based AE reporting on routine MoHCC forms**	X	X	X
**Human resource management**			
**Site-level (facility)**			
**2wT enrolment**		X	X
**SMS-based patient triage**		X	
**In-person reviews**		X	X
**Tracing for no SMS follow-up**		X	X
**Documentation of patient reviews and AEs**	X	X	X
**Hub (Central/MoHCC level)**			
**SMS-based triage**			X
**Refers potential AEs to sites for review**			X
**Calls 2wT men with no SMS contact by Day 7**			X
**Refers 2wT patients for site-based tracing**			X

2wT expansion was initially delayed six months due to COVID-19. Thereafter, a virtual sensitization meeting was held by Zim-TTECH in October 2020, with MoHCC national and District Health Executive structures. With stakeholder buy-in, a phased scale up was planned to include training of site teams (nurses, data clerks, administration) from 3 sites to 32 over 12 months. 2wT was piloted in routine services starting in January 2021, rolling out in February 2021.

#### Tiered approach to 2wT at scale

*Tier 1*: *Patient level*. For patients, any phone that receives and sends SMS messages is sufficient for 2wT. Patients receive 2wT sensitization at MC registration and request to opt-in. Site nurses enrol eligible patients into 2wT and confirm receipt of enrolment message on patient phone before discharge. 2wT patients receive short instruction on responding to 13 days of daily messaging. Normal SMS charges apply. Patients may send free SMS requesting call back. Patient is traced by phone or home visit if no SMS response by day 8.

*Tier 2*: *Site level spokes (facility or outreach setting)*. Routine MC nurses at sites act as 2wT “spokes.” *Spoke* nurses are site-level MoHCC nurses who enrol 2wT patients and interact with patients enrolled at their site via 2wT. They receive a mobile phone, data bundles, and half-day training that includes: use of 2wT Android app, enroling patients, interacting with patients via messages, completing app reports, and syncing patient data. Spoke nurses receive 2wT system-driven “Tasks” from the central hub nurse to trace potential loss to follow-up (LTFU) patients and conduct reviews for those with potential AEs. Spokes receive a “nudge” (reminder) if they do not record subsequent patient follow-up. Site nurses complete all reporting in 2wT system and on routine MoHCC paper forms. 2wT app works offline for enrolment and documentation. 2wT must be synced daily using an Internet connection to ensure patients receive daily messages and to receive/send communication with the Hub.

*Tier 3*: *Central level Hub*. 2wT has a central Hub nurse to triage patients across different 2wT sites. Central Hub location employs an Android phone with a sim card to send and receive SMS (gateway) that is linked to a desktop PC with a stable internet connection to allow communication between 2wT system, Hub, and spokes. Hub nurse monitors up to 500 patients a day across all sites using the 2wT web interface, reviewing daily patient reports and providing patient reassurance or education via 2wT. To support Hub efficiency, a specific red icon (“!”) draws attention to all patients that report a potential AE. Hub triages those in need of referrals to care and pushes out a 2wT-based system “Task” to site spokes for follow-up. Hub also creates tracing tasks for patients without a response by day 8. Site nurses are required to close tasks by completing an outcome report on subsequent follow-up in-person reviews or tracing efforts. Hub nurse provides scheduled on-site supervision and routine mentoring of site nurses via a WhatsApp 2wT Support Group. Routine ZAZIC oversight support and system monitoring ensures quality care and builds team confidence.

#### Study participants

Comparison between 2wT from RCT and routine scale-up 2wT draw upon previously published RCT data procedures and outcomes [12,18]. For the scale-up period, participants included all adult males over age 18 that received an MC in the ZAZIC routine VMMC program, either 2wT-based or in-person post-operative follow-up, between January 2021, and October 2021.

#### Routine study sites and recruitment

2wT men were enrolled as part of routine MC service delivery across 32 VMMC sites. Specific demand creation efforts supported 2wT recruitment, including posters and branded 2wT face masks. Enrolments were done during pre- and/or post-operative 2wT education, depending on the preferences and patient flow of each MC setting. MC patients received no specific 2wT consent as part of this routine service delivery follow-up approach. 2wT participants agreed to respond to SMS follow-up for 13 days on their phones. No compensation was offered to offset patient texting costs.

#### Data collection

Aggregate data on MC enrolments of adult male patients (ages ≥18), AEs, and in-person visits were gathered from routine MoHCC registers as part of routine reporting across 37 MC sites. Additional information on 2wT patient enrolment, SMS interactions, in-person follow-up, and referrals were ascertained from Medic’s 2wT database in 32 sites as part of this quality improvement activity.

#### Data analysis

The primary outcomes of interest were cumulative notifiable AE rates calculated as: (# moderate + severe AEs)/ (total # MCs) per group (RCT, 2wT routine, and routine in-person follow-up). Secondary outcomes included: # with ≥1 daily SMS response; spontaneous SMS (patients who sent a free text message, not in response to the daily prompt); patients who reported a potential AE; and LTFU (no contact within 14 days). No SMS contact by day 8 is reported only for 2wT in routine settings. Cumulative rate of any moderate or severe AE, and in-person visits were compared using STATA 15 immediate commands for aggregate data (StataCorp, College Station, TX), reporting 2-sided p-values from Fisher’s exact test with Wilson 95% confidence intervals (CI).

#### Ethics

The ethics committee of the University of Washington (UW) internal review board (IRB) instructs UW researchers to complete the Human Subjects Research Worksheet to self-determine if the proposed project meets the definition of research and therefore requires IRB review. This study using secondary aggregate data did not meet the definition of *research*, and therefore did not require UW IRB review.

## Results

### Characteristics of 2wT patient behaviours in the routine setting

**Fig 6:**
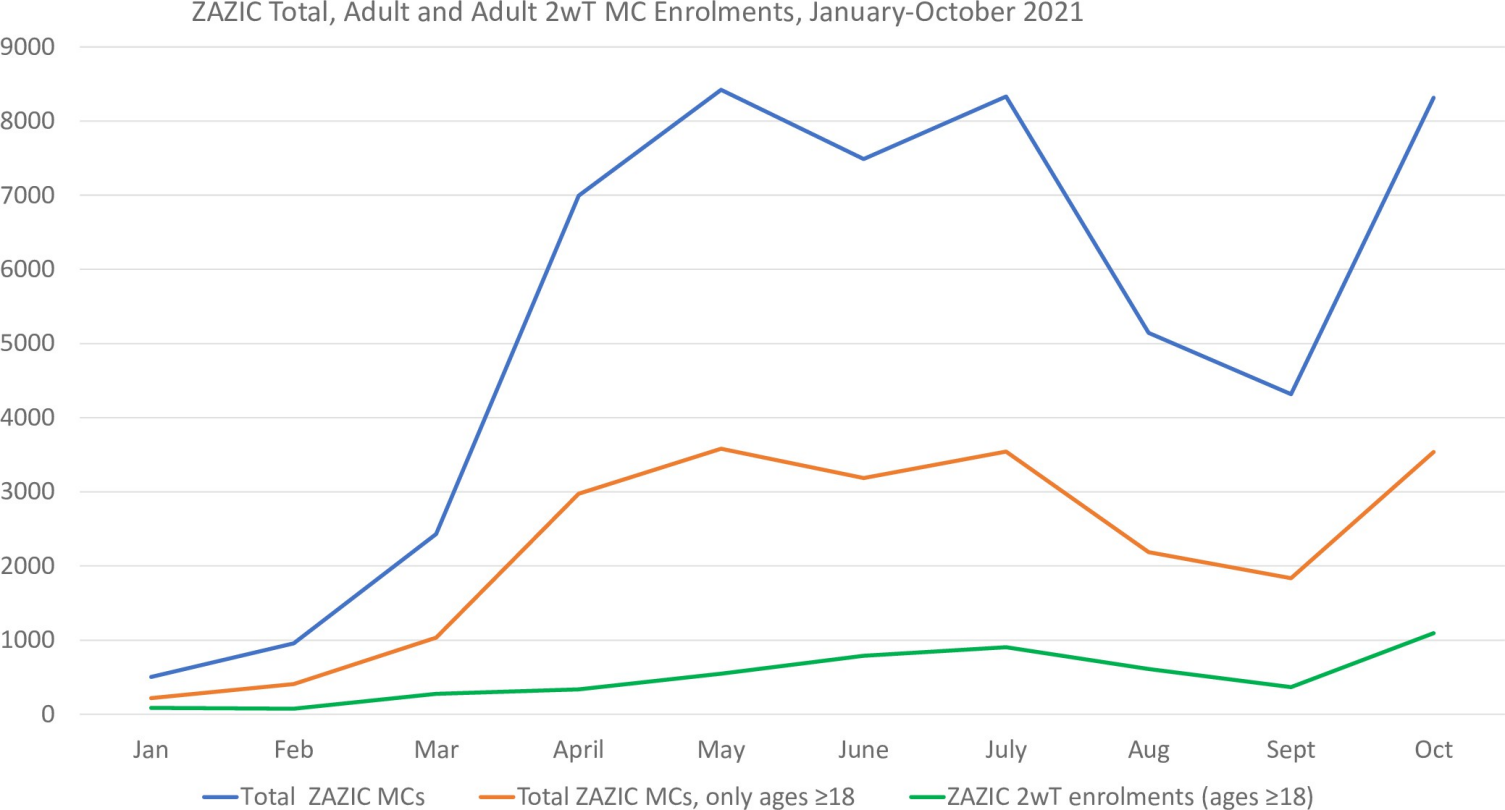
ZAZIC MC enrolments January 2021-October 2021, by: total MCs (ages ≥15), adult MCs (ages ≥18), and 2wT enrolments (ages ≥18).

2wT enrolment and expansion increased as COVID-19 restrictions eased (Fig 6). In the period from January 2021 to October 2021, 17417 men received in-person MC follow-up across all 37 sites while 5084 patients were followed-up by SMS (Table 2) from 32 sites. Of 5084, there were 5078 unique phone numbers, accounting for three sets of enrolled brothers who shared phones. Among the 5084 2wT participants, 3609 (71.0%) replied to at least 1 daily SMS and 1,475 (29%) were followed up (traced) for no SMS contact by day 8. Of those traced on day 8, patient phone or home tracing found 1389 (94.2%) who did not need additional follow-up, 20 (1%) needed care, and 66 (4.5%) were not reached. Of all 5084, 630 (12.4%) patients reported at least one potential AE, and 325/5084 (6.4%) received individualized 2wT-based telehealth from the Hub (reassurance, reminders on wound care, and hygiene advice). Of the 325, 261 (80.3%) were subsequently cleared of having an AE via SMS or phone interaction while 64 (19.7%) were referred to site-level spokes for in-person review. Thirty-two referred patients (50%) attended a visit. An additional 34 2wT patients attended a follow-up visit spontaneously without requesting help via SMS first. Of all 2wT patients, only 4 (0.08%) notifiable AEs (1 severe and 3 moderate) were identified and reported. Within 14 days, 99.2% men had at least one follow-up contact with a nurse, 93.2% via SMS, 4.8% via voice call and 1.2% via an in-person visit.

**Table 2:** Comparison of MC outcomes: 2wT RCT vs. 2wT routine; 2wT routine versus in-person routine.

	(a)	(b)	(c)	(d)	(e)
	2wT RCT* # (%)	2wT Routine # (%)	p-value	In-person Routine	p-value
MC Patients	362	5084	-	17417	
Enrolling sites	2	32	-	37	
Notifiable AEs	6 1.9% (0.7, 3.6)	4 0.08% (0.03, 2.0)	<0.001	6 0.03% (0.02, 0.08)	0.248
In-person visits	82 22.7% (18.6, 27.2)	66 1.1% (1.0, 1.6)	<0.001	11669 67.0% (66.3, 67.7)	<0.001
Lost to follow-up (LTFU)	25 6.9% (4.7, 10.0)	40 0.8% (0.6, 1.1)	<0.001	5748 33.0% (32.3, 33.7)	<0.001
≥ 1 daily SMS response	334 92.5% (89.0, 94.6)	3609 71.0% (69.7, 72.2)	<0.001	-	-
Spontaneous SMS	256 70% (65.8, 75.2)	1718 33.3% (32.5, 35.1)	<0.001	-	-
Potential AEs	156 43.1% (38.1, 48.2)	630 12.4% (11.5, 13.3)	<0.001	-	-
No SMS contact by day 8	-	1475 29% (29.9, 32.5)	-	-	-

*RCT results reported previously [18]. Routine 2wT and in-person enrolments from January 2021 to October 2021. p-values from Fisher’s exact test using STATA immediate commands. (c) p-value from 2wT in RCT (a) compared to 2wT in routine (b). (e) p-value from routine 2wT (b) versus in-person follow-up (d) in routine settings. 95% CI in parentheses.

### 2wT-based outcomes: RCT and routine settings

As compared to the RCT setting (Table 2, columns a and b), in the routine context, 2wT ascertained fewer AEs, with AEs rates decreasing from 1.9% (95% CI: 0.7, 3.6) to 0.08% (95% CI: 0.03, 2.0), respectively (p<0.001). HCW workload also reduced from the RCT to routine setting: only 1.1% (95% CI: 1.0, 1.6) of routine 2wT men had a documented in-person visit versus 22.7% (95% CI: 18.6, 27.2) during the RCT (p<0.001). In the RCT, 92.5% (95% CI: 89.0, 94.6) responded at least once over 13 days, but the 2wT response rate fell to 71.0% (95% CI: 69.7, 72.2) at scale (p<0.001). Prevention of LTFU improved during 2wT at scale: only 0.8% (95% CI: 0.6, 1.1) of 2wT men had no contact by 14 days as compared to RCT at 6.9% (95% CI: 4.7, 10.0) (p<0.001).

### Scale-up outcomes: Comparison of 2wT to routine in-person follow-up

During the January 2021-October 2021 period (Table 2, columns b and d), AE rates were similar across 2wT [0.08% (95% CI: 0.03, 2.0)] and routine follow-up [0.03% (95% CI: 0.02, 0.08)] (p = 0.0248). The four 2wT patients with AEs (three moderate and one severe) all exchanged texts with the Hub nurse and were referred to care before AE identification (Table 3). There were more severe AEs among routine care men (4 severe and 2 moderate).

**Table 3:** Reported AEs among adult patients across ZAZIC sites, January-October 2021.

Follow-up type	Severity	AE Timing-Type	# Days Post Op
2wT			
	Severe	B-BL	1
	Moderate	B-BL	2
	Moderate	B-BL	3
	Moderate	C-IN	17
In-person			
	Severe	B-BL	1
	Severe	B-BL	1
	Severe	B-BL	3
	Severe	C-IN	19
	Moderate	B-BL	1
	Moderate	C-IN	8

Timing of AEs: A = Intra-operative or immediately postoperative (prior to discharge from clinic); B = After discharge and up to 7 days post-operative; C = Greater than 7 days post-operative. AE types: BL = Bleeding; IN = Infection.

HCW workload was dramatically lower with 2wT: only 1.1% (95% CI: 1.0, 1.6) of 2wT versus 67.0% (95% CI: 66.3, 67.7) of routine follow-up men had an in-person review. Among 2wT patients, 31.2% (95% CI: 29.9, 32.5) had no contact by day 8, but only 0.8% (95% CI: 0.6, 1.1) of 2wT men were ultimately reported as LTFU as compared to 33.0% of men with expected in-person follow-up (95% CI: 32.3, 33.7; p<0.001).

## Discussion

Despite the challenges of MC service delivery during the COVID-19 pandemic, 2wT scaled successfully from the RCT to routine settings. In both the RCT and the routine setting, 2wT-based follow-up assured patient safety while dramatically reducing provider workload. Patient follow-up rates and AEs were similar in either follow-up method, suggesting equivalence in assuring healthy post-operative healing. Although 2wT enrolment was slower than expected, the advantages of 2wT for MC patients and providers is clear. In response to both COVID-19-created opportunities to advance digital health innovation [20–22] and recent shortfalls in global MC targets [23], MC policy and programs should support 2wT expansion. Several key aspects of this intervention’s success and potential challenges for scale-up merit discussion.

### 2wT successes

First, unlike simpler bi-directional texting [24–26], this 2wT system adds several value-added features. 2wT includes a daily care prompt to encourage men to observe their healing and engage directly with an MC nurse to ask questions or request a call back. The centralized Hub nurse provides personalized telehealth for each patient, triaging those that need follow-up by referring to MC spoke nurses in local healthcare centres. For men who do not respond to daily texts by day 8, a 2wT system-generated alert ensures that the Hub nurse follows-up potential LTFU with calls to next of kin or referrals to site-based tracing. 2wT generated alerts at site level close referral loops for in-person reviews or tracing, confirming that men receive in-person care when needed. Patient phone numbers are confirmed via visual receipt of the enrolment text on the patient phone. This authentication process also provides confirmation of MC patients, adding a layer of verification of MC productivity.

Second, in routine settings, 2wT maintains the quality of care while dramatically reducing in-person visits. Although underreporting of AEs is possible [8], similar AE rates between 2wT and routine follow-up groups suggests safety for either approach. All reported AEs from 2wT men followed an initial “potential AE” SMS or interaction with the 2wT nurse, suggesting that men were both comfortable and confident to seek care via 2wT if they were experiencing a challenge or potential complication during their healing process. Among 2wT patients who did not respond to a daily text by day 8, phone and in-person follow-up did not result in identification of additional AEs, suggesting that lack of SMS response was largely due to lack of patient concerns. Although in-person follow-up rates are often reported at nearly 100%, actual follow-up may be far lower [10]. In this period, approximately 70% of both 2wT and in-person patients had follow-up contact. However, with similar assurances of patient healing, the workload reduction of benefits of 2wT is a distinct advantage at scale.

Third, 2wT may provide an early warning system for issues in quality service delivery. 2wT patient and provider interaction identified a challenge among circumcised patients that was undetected or unreported among routine MC men: delayed dissolution of suture materials. A total of 23 men (5.3%) either texted or requested a call between post-operative days 9 to 40 with concerns about remaining intact sutures. Although review resulted in only mild, non-notifiable AEs, these cases triggered an investigation into the suture material and commodity review. The investigation identified suture materials in consumable packs nearing expiration or already expired, potentially signalling their reduced quality. MC teams were alerted, and the kits were disposed of according to MoHCC protocols for expired medicines and equipment.

### 2wT challenges

Although enrolment target was set for 60% of eligible men, only 28.7% of 2wT-eligible men were enrolled during this initial scale-up period. Several program-level factors likely contributed. First, urban centres experienced high COVID-19 cases, slowing MC services and shifting service delivery to outreach facilities with weaker network coverage. Second, MCs were also performed among groups without phones, such as prisoners. Lastly, most MCs were performed among boys ages 15–17 during this period, before 2wT was approved for guardians of minors in 2022. Moreover, at facility level, adoption of 2wT is highly dependent on providers’ willingness to enrol patients. 2wT patients still require completion of MoHCC paper forms, creating redundancy, added work, and potential data duplication. Providers may also be reticent to have additional oversight by Hub nurse and ZAZIC QA teams.

2wT is not unique in identifying digital health scale-up barriers [27,28]. To support wider scale-up and sustainability in Zimbabwe, several improvements are needed. First, 2wT practice-based training, on-site supervision, and consistent mentoring are needed. This will help ensure 2wT SOP adherence and consistency while expanding to more districts and increasing 2wT access. Second, enhancing 2wT system capacity and patient SMS costs could be assumed by ZAZIC or MoHCC, reducing patient participation costs. Third, to increase patient demand, ZAZIC is launching robust messaging of 2wT through pamphlets, posters, and training of demand creation cadres in recruiting and messaging. Fourth, although 2wT has proven safe for patients, international MC funders still require in-person visits for global reporting, creating fear that wide deployment of 2wT could create tension between donors and MoHCC policy. Supportive 2wT policy must be in place to support scale-up, including allowing enrolment for minors and guardians to aid uptake. Lastly, to avoid duplication and support workload reduction, reporting redundancies across MoHCC paper forms and 2wT must be swiftly addressed.

### Limitations

Although the short 14-day follow-up period reduces concerns of phone loss or number changes, sixty-nine men responded to daily texts from numbers that were not entered into the 2wT system. These responses could not be linked to a specific patient, muddling the precision of response rates. AEs may have been misidentified or undocumented, potentially underreporting notifiable AEs in any group. 2wT should be considered as a critical tool to increase the quality of MC services and reduce workload but will not solve typical barriers to follow-up such as mobile populations, inaccessible roads, and overstretched VMMC teams.

## Conclusions

There is strong evidence that 2wT scale-up in routine settings appears advantageous for both patients and nurses, reducing workload and maintaining patient safety. In the context of COVID-19, 2wT proved even more advantageous: 2wT reduced contact between service providers and patients in accordance with WHO guidelines on COVID-19 prevention. Despite its slow expansion, 2wT scaled more swiftly in 2022. By April 2022, 2wT enroled over 10,000 ZAZIC patients, up to 80% of eligible clients per week, a sign of growing 2wT momentum.

Globally, proven systems like 2wT still face substantial organizational, institutional, and global obstacles to potential impact, mirroring experiences from, and perspectives on, digital health interventions in low- to middle-income countries (LMIC) [29,30]. First, lack of coherence and cooperation between Ministries, donors, and implementing partners remains a major challenge for digital health to deliver maximum benefits to vulnerable communities. Ministries need support to foster digital health sustainability, calling for significant investments from capacity strengthening through supportive infrastructure. Second, policy and governance must keep pace. Digital health innovations are occurring swiftly, but efforts lag to standardize Ministry policy and align donor support for sustained progress and impact [31]. Third, emphasis on national-level systems or interoperability concerns should not prevent scale-up of evidence-based innovations. While national adoption of Health Information Systems (HIS), or electronic medical records (EMR) offer considerable promise, few have successfully scaled [27,32]. Immediate Provincial or District adoption of 2wT could serve millions of MC patients in comparison to years needed to develop, deploy, and optimize a national EMR system. Where possible, digital health innovations like 2wT could swiftly operationalize data exchange with HIS or EMR (a lower technical bar based on offline data transfers and syncing) without needing complex, and quickly out of date cross coding, or interoperable systems. WHO, donors, and Ministries should revise MC follow-up policies and support rapid expansion of 2wT across MC programs. Consideration of 2wT-based telehealth for other contexts could safely expand efficiency gains from MC to other outpatient or acute-care contexts.

## Supporting information

S1 Data ZAZIC aggregate data by month, January 2021-October 2021. ZAZIC Total MCs, Adult MCs, and Adult 2wT MC Enrolments, January-October 2021
(XLSX)
